# Sow reproductive and progeny growth performance when fed *Pichia guilliermondii* yeast postbiotic: systematic review and meta-analysis

**DOI:** 10.1093/tas/txae137

**Published:** 2024-09-14

**Authors:** Clementine Oguey, Morgan T Thayer

**Affiliations:** ADM International SARL, 1180 Rolle, Switzerland; ADM Animal Nutrition, Quincy, IL 62301, USA

**Keywords:** litter size, meta-analysis, *Pichia* yeast, postbiotic, progeny performance, sow performance

## Abstract

Previous research suggested that feeding sows with a product containing an inactivated strain specific *Pichia guilliermondii* yeast postbiotic (**PG**; Citristim, ADM Animal Nutrition, Quincy, IL) has the potential to support fecundity, and progeny performance at birth, weaning, and after weaning. To summarize these effects, a systematic review followed by a meta-analysis was carried out to determine the effects of feeding sows with PG during gestation and lactation on reproductive the performance of sows and the growth of progeny after weaning. All experiments included were randomized trials reporting side-by-side comparisons of an appropriate control (**CON**) and the CON with the inclusion of PG. The effects of PG inclusion in sow diets were evaluated using the raw mean difference and effect size calculations. Analysis included seven trials for sow reproductive and litter performance until weaning, and eight trials for progeny performance after weaning. The risk of publication bias was assessed by funnel plots. In the case of publication bias, the Trim and Fill method was used. Heterogeneity was assessed using *I*^2^ statistics. Sows fed PG during gestation and lactation had more piglets born alive (BA), BA + stillborn, and BA + stillborn + mummies (*P* < 0.001). The individual birth weight of the piglets was not affected by the supplementation (*P* = 0.835). As a result, litter weight at birth was greater in sows-fed PG (*P* < 0.001). Piglets born from PG-fed sows tended to be weaned 0.34 d younger than those from CON-fed sows (*P* = 0.060). Twenty-one-day adjusted pig weight at weaning tended to be lighter by 0.122 kg in the PG sow group (*P* = 0.069); however, litter weight at weaning adjusted to 21 d remained similar across groups (*P* = 0.516). The number of piglets weaned and mortality-adjusted number of piglets weaned per sow were greater in PG than in CON sows (*P* < 0.023). A carryover effect was observed for progeny of PG-fed sows after weaning. Piglets born from PG-supplemented sows had greater weight gain (*P* = 0.030) and tended to have a better survival rate (*P* = 0.055) until the end of the nursery phase. These results indicate that feeding PG to sows during gestation and lactation consistently and significantly improves not only the performance of sows at farrowing but also performance of the progeny after weaning.

## Teaser

A meta-analysis showed that feeding *Pichia guilliermondii* yeast postbiotic to sows during gestation and lactation improved sow fecundity and increased litter size at weaning.

## Introduction

Over the past three decades, pig breeding strategies have aimed to increase litter size and minimize piglet mortality. These improvements in sow performance parameters are well known to decrease not only production costs but also decrease the environmental footprint per kg of pork produced (Knap et al., 2023). Dietary interventions are one of the available strategies to further increase sow productive performance. Among them, biotics are defined as nonnutritive components that cover the concepts of probiotics, prebiotics, synbiotics, and postbiotics.

The appearance of the term postbiotics has increased dramatically in published literature since 2020. In contrast to live microorganisms known as probiotics, postbiotics are defined as the “preparation of inanimate microorganisms and/or their components that confers a health benefit on the host” (Salminen et al., 2021). Many different postbiotics are currently used in feed for livestock, among which are yeast-based products. Previous research suggested that heat-inactivated *Pichia guilliermondii* yeast, a co-product of citric acid fermentation, had the potential to promote improved reproductive performance of sows (Bass et al., 2019; Thayer et al., 2023). However, such products generally suffer from a lack of consistent effects on animal performance. Therefore, the intent of this study was to conduct a systematic review and a meta-analysis of the effects of an inactivated *P. guilliermondii* yeast postbiotic (**PG**; Citristim, ADM Animal Nutrition, Quincy, IL) supplemented to sows during gestation and lactation, on the reproductive performance of sows, and growth performance of piglets prior to and after weaning.

## Materials and Methods

This report contains data from previously conducted research. No additional animals were used to perform this work, therefore no animal care and use protocol was necessary.

### Data Sources and Description of the Databases

The first step in conducting a meta-analysis is to run a systematic review of all reports available on the concerned topic to identify relevant literature. The literature search and study selection were performed following the preferred reporting items for systematic reviews and meta-analyses (PRISMA) statements (Page et al., 2021). The meta-analysis was conducted to assess the effect of feeding sows an inactivated strain-specific *P. guilliermondii* yeast product (PG; Citristim, ADM Animal Nutrition, Quincy, IL) on the productive performance of sows and their progeny after weaning. To retrieve published and unpublished reports, a literature search was performed through online academic databases including PubMed, Google Scholar, Mendeley, Web of Science, and ADM company’s internal trial database. The search strategy included the words “Citristim” OR “Pichia” OR “*P. guilliermondii*” AND “sow”, and included trials finalized until the end of December 2023. Authors independently screened relevant research reports identified by the search strategy. A total of 19 reports were initially identified through the literature search for sow and postweaning performance combined.

Next, an exclusion phase was completed to remove reports that did not comply with the scope of the work or did not meet quality requirements. Of the 19 reports identified, there were reports excluded because they were present in duplicate (10), they deviated from the protocol (1), or they did not meet eligibility criteria (2). Not meeting eligibility criteria could include if PG was only fed during lactation and not gestation and lactation, or if there was a lack of clarity in raw data.

After exclusion criteria were applied, six reports remained for evaluation and raw data were available for all of them. Two reports presented only sow performance (Thayer et al., 2023 and Unpublished Canadian trial), whereas four reports presented both sow and postweaning piglet performance outcomes. The manuscripts published by Bass et al. (2019; 2013), Thayer et al. (2020; 2022), Unpublished French trials, and Hernandez et al. (2021) shared data from the same report covering the sow and postweaning piglet performances, respectively.

Hernandez et al. (2021) reported 2 sow studies each paired with postweaning piglet performance. Study 1 (sows and piglets) was excluded because PG was fed only during lactation. In study 2, the postweaning piglet performance was included in the meta-analysis but the sow performance was excluded from the database because there was a lack of clarity in the raw data.

In total, there were seven trials organized in 5 reports presenting results on sow performance and eight trials organized in four reports for piglet performance after weaning. Each trial is the comparison of CON or PG fed sows, or their piglets in the nursery, at the same dose of PG, the same method of weighing the pigs, the same experiment within the report, or the same block of the experiment. Characteristics of the studies included in the analyses are given in Tables 1 and 2.

**Table 1.** Description of the comparisons included in the sow meta-analysis^*^

Trial	Housing condition	PG sow dose, kg/t	CON Sows, *n*	PG Sows, *n*	Country	Reference
Trial 1^†^	University	1	32	33	US	Bass et al., 2019
Trial 2^†^	University	2	32	33	US	Bass et al., 2019
Trial 3^‡^	Farm	1.5	195	200	US	Thayer et al., 2023
Trial 4^‡^	Farm	1.5	105	106	US	Thayer et al., 2023
Trial 5	Farm	1	261	272	US	Thayer et al., 2020
Trial 6	Experimental farm	1	25	26	FR	unpublished
Trial 7	Farm	1	454	350	CA	unpublished

^*^A total of 7 trials were used in this meta-analysis to determine the sow and litter performance effects of feeding sows a product containing *P. guilliermondii* yeast postbiotic (PG; Citristim, ADM Animal Nutrition, Quincy, IL) during gestation and lactation.

^†^Trials 1 and 2 occurred in the same study with two doses of PG. Each treatment group was compared to control fed sows.

^‡^Trials 3 and 4 occurred in the same study. Sows were separated into two treatment groups. The 395 sows had their litters weighed on a whole litter basis divided by the number of pigs in the litter. The 211 sows had their litters weighed on an individual piglet basis.

**Table 2.** Description of the comparisons included in the post weaning meta-analysis^*^

Trial	Housing condition	PG sow dose, kg/t	CON Piglets (Pens), *n*	PG Piglets (Pens), *n*	Duration, days	Country	Reference
Trial 1^†^	University	1	112 (16)	112 (16)	35	US	Bass et al., 2013
Trial 2^†^	University	2	112 (16)	112 (16)	35	US	Bass et al., 2013
Trial 3^†^	University	1	92 (16)	92 (16)	35	US	Bass et al., 2013
Trial 4^†^	University	2	92 (16)	92 (16)	35	US	Bass et al., 2013
Trial 5	Farm	1	627 (30)	627 (30)	42	US	Thayer et al., 2022
Trial 6^‡^	Experimental farm	1	55 (4)	50 (5)	42	US	Hernandez et al., 2021
Trial 7 ^‡^	Experimental farm	1	93 (12)	88 (13)	42	US	Hernandez et al., 2021
Trial 8	Experimental farm	1	28 (7)	28 (7)	35	FR	unpublished

^*^A total of 8 trials were used in this meta-analysis to determine the effects of feeding sows a product containing *P. guilliermondii* yeast postbiotic (PG; Citristim, ADM Animal Nutrition, Quincy, IL) during gestation and lactation on progeny performance after weaning.

^†^Trial 1 through 4 are from the same research report with two separate experiments included. Trial 1 and 2 occurred in experiment 1 with two doses of PG fed to the sow. Trial 3 and 4 occurred in experiment 2 with two doses of PG fed to the sow. Pens of pigs fed the nursery control diet were used in this meta-analysis and pens of pigs fed PG in the nursery were excluded.

^‡^Trial 6 and 7 are from the same research report with two separate and unequal blocks of an experiment (study 2) included.

Trials were included only if they were randomized trials that reported side-by-side comparisons of sows fed a basal diet (**CON**) compared to sows fed the same diet supplemented with PG during gestation and lactation over one reproductive cycle. Those trials had to report data on sow performance at birth and weaning of offspring, or on the performance of piglets after weaning known as the carryover effect. In addition, the sample size of experimental groups, the mean of each parameter and standard deviation (**SD**) were necessary information for trials to be included in the database. Raw data were used for thev calculations described. Trials were excluded if PG was fed in combination with another product.

Evaluation of data were divided into two sections which include sow performance at farrowing and weaning, and piglet performance after weaning. The first section, evaluation of sow performance, was assessed through total number of piglets born (born alive + stillborn + mummies; **BA + SB + Mums**), number of born alive + stillborn piglets (**BA + SB**), number of piglets born alive (**BA**), litter size before and after cross-foster and at weaning, litter weight at birth and weaning, average piglet weight at birth and weaning, weaning age, and proportion of piglets lighter than 900 g at birth within a litter.

Adjusted litter size at weaning was calculated considering survival and number of pigs BA, according to Equation 1, with mortality being calculated as the sum of mortalities pre and post-cross-foster. Twenty-one day adjusted weaning weight was calculated by applying Equation 2. Finally, 21-d adjusted litter weight was obtained by multiplying 21-d adjusted pig wean weight by the number of piglets weaned.


$$\begin{array}{l} mortality\;adjusted\;nb\;weaned\;(n)=(1-mortality)*number\;BA \end{array}$$ (1)

$$\begin{array}{lll} 21\;d & \;adjusted\;pig\;wean\;weight\;(kg)\; & =piglet\;weaning\;weight+ADG*1.1*(21-wean\;age) \end{array}$$ (2)

The second section evaluated the carryover effects of feeding PG to sows during gestation and lactation on piglet performance after weaning using piglet weaning weight, average daily gain (**ADG**), final pig weight at the end of the nursery, and mortality during the postweaning phase.

### Effect Size Estimates

Effect size gives a quantitative measure of the magnitude of the difference between two groups. The evaluation of the effect of PG on the outcomes was performed using the Metafor package (version 4.4.0; Viechtbauer, 2010) of the R Statistical Software Program (version 4.0.3; R Core Team, 2020). The number of observations and standard deviation were used to calculate an estimate of within-study variance. The reciprocal of within-study variance was used to weigh the contribution of each study to the overall estimate of effect size and variability. The model was fitted using the restricted-maximum likelihood estimation. If this estimation did not converge, then the maximum likelihood estimation was used. Raw mean difference (**RMD**) was used to display effect size of continuous outcomes. For piglet mortality after weaning, binary data approach was applied, and the odd’s ratio (**OR**) was considered. Forest plots were used for each study to visually display the estimated effect size with its 95% confidence interval. Effect of PG on RMD and OR was declared significant when *P *≤ 0.05 and tendency when 0.05 < *P *≤ 0.10.

### Evaluation of Risk of Bias and Heterogeneity

**Figure 1.**
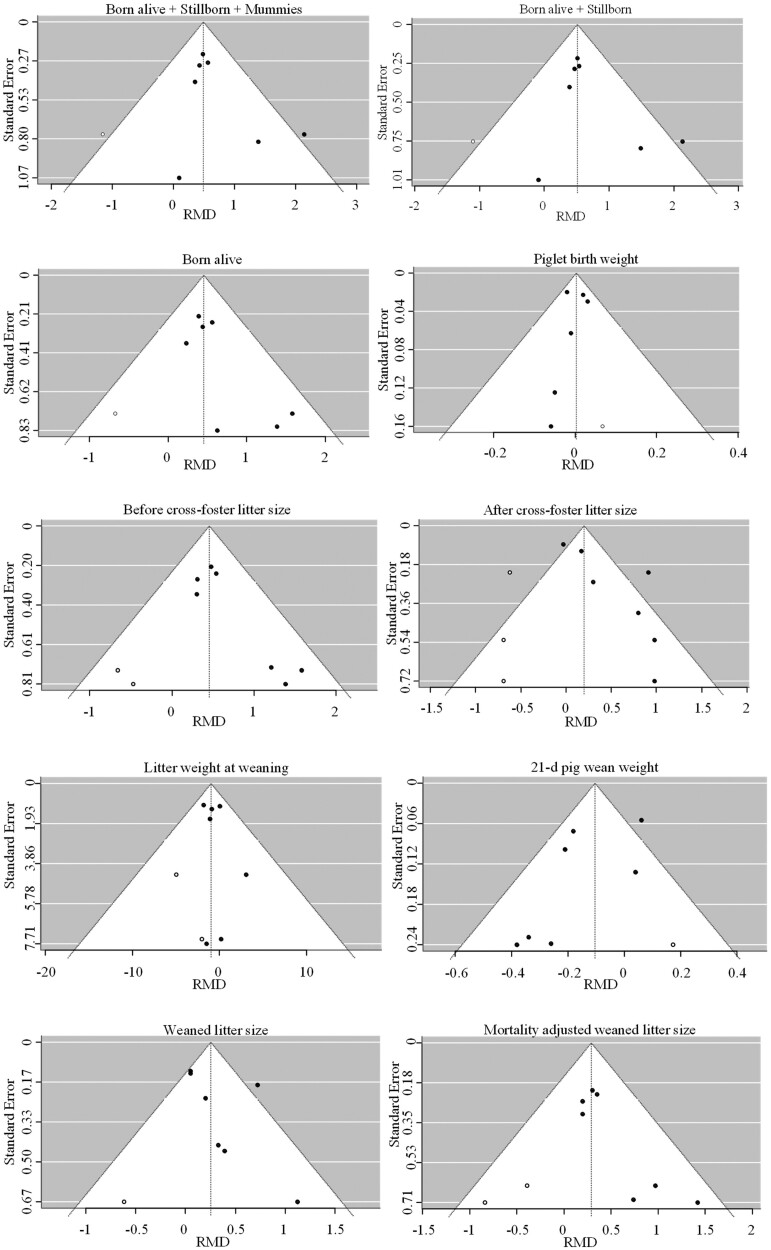
Funnel plots of outcomes related to sow performance at birth and at weaning (□: 95% confidence interval region; ●: observed studies; ○: imputed estimated missing studies).

In research, trials resulting in positive outcomes tend to be published more often than those that show no effect or a negative outcome. This publication bias can risk an overestimation of the effect of an intervention and needs to be evaluated. In the present work, the risk of publication bias was evaluated graphically with funnel plots (Figure 1). The Trim and Fill method (Duval and Tweedie, 2000) was used to estimate the number of missing studies that might exist and what the effect might be on the outcome. R0 estimator was considered and tests the null hypothesis that the number of missing studies is zero. Risk of publication bias was considered significant when *P* ≤ 0.05.

The last step consisted of assessing whether heterogeneity was present. A meta-analysis aims at quantifying a global effect of an intervention which assumes that all studies are homogeneous. Heterogeneity means that studies have different characteristics that influence the effect of the intervention. Heterogeneity was evaluated by use of the *I*^2^ statistic (Higgins et al., 2003). An outcome with *I*^2^ > 0.40, and *P *< 0.05 was considered as substantially heterogeneous.

## Results

### Effect of PG on Performance of Sows Until Weaning

The seven comparisons included in this analysis involved 2092 sows in total. Inclusion of PG in the sow’s diet varied from 1 to 2 kg/metric ton. The effects of PG on litter performance from birth to weaning are detailed in Table 3. Forest plots are given in Supplementary Appendix 1. Sows fed PG exhibited greater fecundity compared to non-supplemented animals, as shown through more piglets born (RMD for BA + SB + Mums, BA + SB, and BA of 0.54, 0.56, and 0.49, respectively; *P* < 0.001). Piglet birth weight and proportion of light piglets were similar across the groups (*P *< 0.835). Litter weight at birth was 814 g heavier with sows being fed PG vs. CON (*P* < 0.001). No heterogeneity was observed for any of the farrowing performance outcomes listed above (Table 3; *I*^2^*P* > 0.05). A risk of publication bias was identified for BA + SB + Mums, BA + SB, BA, and piglet birth weight; however, the Trim and Fill method estimated only 1 missing study for each outcome and was considered not significant (Figure 1; test for null hypothesis *P* < 0.250).

**Table 3.** Effect of *P. guilliermondii* yeast postbiotic fed to sows on litter performance from birth to weaning^*^

Parameter	Control		Effect size calculation				Heterogeneity	
	Mean	SE	RMD	SE	(95% CI)	*P*-value	*I* ^*2,*^ ^†^	*P*-value
BA + SB + Mums,^‡^*n*	14.64	0.37	0.54	0.13	(0.28; 0.80)	<0.001	0	0.423
BA + SB,^$^*n*	14.33	0.40	0.56	0.13	(0.30; 0.82)	<0.001	0	0.383
BA,^¶^*n*	13.39	0.33	0.49	0.13	(0.24; 0.74)	0.001	0	0.636
Litter birth weight, kg	18.614	0.742	0.814	0.248	(0.329; 1.300)	0.001	14.4	0.626
Piglet birth weight, kg	1.413	0.018	0.003	0.15	(−0.025; 0.032)	0.835	12.6	0.654
Light weight piglets,^**^ %	9.99	1.87	0.75	0.95	(−1.11; 2.60)	0.431	24.8	0.292
Before cross-foster, *n*	12.88	0.20	0.51	0.12	(0.27; 0.75)	<0.001	0	0.521
After cross-foster, *n*	13.08	0.21	0.44	0.18	(0.10; 0.79)	0.012	77.8	<0.001
Weaned, *n*	11.19	0.25	0.28	0.13	(0.02; 0.55)	0.023	58.1	0.035
Litter weight at weaning, kg	66.67	0.82	−0.86	0.59	(−2.01; 0.29)	0.143	0	0.879
Piglet weight at weaning, kg	5.902	0.091	−0.221	0.068	(−0.355; −0.088)	0.001	54.2	0.043
Age at weaning, d	19.91	0.45	−0.34	0.18	(−0.69; 0.01)	0.060	87.0	<0.001
21-d pig wean weight,^††^ kg	6.20	0.07	−0.122	0.067	(−0.253; 0.009)	0.069	55.8	0.026
21-d litter wean weight,^††^ kg	69.29	1.65	0.50	0.78	(−1.02; 2.03)	0.516	31.0	0.320
Adjusted weaned,^‡‡^*n*	10.92	0.29	0.35	0.12	(0.12; 0.57)	0.003	0	0.653

^*^A total of 7 trials were used in this meta-analysis to determine the sow and litter performance effects of feeding sows a product containing *P. guilliermondii* yeast postbiotic (PG; Citristim, ADM Animal Nutrition, Quincy, IL) during gestation and lactation. Data from five studies were used for the percentage of light weight piglet calculations. Data from six studies were used for litter and piglet birth weight calculations. All other parameters were calculated using data from seven studies.

^†^I^2^ can be interpreted as the proportion of total variation observed between the trials attributable to differences between trials rather than sampling error (chance).

^‡^Number of pigs born alive + stillborn + mummies.

^$^Number of pigs born alive + stillborn.

^¶^Number of pigs born alive.

^**^The proportion of piglets lighter than 900 g at birth within a litter.

^††^Individual pig and litter wean weights were adjusted to a common 21 d of age at weaning.

^‡‡^The number of pigs weaned were adjusted by multiplying the number of pigs born alive by preweaning mortality.

RMD, raw mean difference; 95% confidence interval.

In line with results observed on farrowing performance, the number of piglets per sow before cross-foster was greater by 0.51 piglets per sow in PG compared to CON (*P* < 0.001). No heterogeneity was identified (*I*^2^ = 0; *P* = 0.521), and with number of missing studies estimated as 2 (Figure 1; test for null hypothesis *P* = 0.125), the risk of publication bias was not significant for litter size before cross-foster.

However, after cross-foster, the benefit of PG over CON on litter size was reduced to 0.44 piglets per sow (*P* = 0.012). Significant heterogeneity was identified (*I*^2^ = 77.8; *P < *0.001). It was estimated that three studies were missing (Figure 1; test for null hypothesis *P* < 0.07) suggesting a trend for risk of publication bias. Correction of risk of bias would result in similar litter size post cross-foster between PG and CON (corrected RMD = 0.20; *P* = 0.31).

The number of piglets weaned, and mortality-adjusted number of piglets weaned per sow were greater respectively by 0.28 and 0.35 piglets per sow in PG compared to CON (*P *< 0.023). Piglets tended to be weaned 0.34 d younger when from sows fed PG compared to CON (*P* = 0.060). Piglet weaning weight was 221 g per pig less in the PG group (*P* < 0.001). The 21-d adjusted piglet weights at weaning tended to be lighter when from sows fed PG (*P* = 0.069). On the contrary, litter weight at weaning, and 21-d adjusted litter weights were not different with PG supplementation (*P* > 0.143).

The number of missing studies was estimated as 1 for weaned litter size (Figure 1; test for null hypothesis *P* < 0.250), and two for litter weight at weaning and mortality-adjusted weaned litter size (Figure 1; test for null hypothesis *P* < 0.125). Therefore, a risk of publication bias was identified for those performance outcomes at weaning; however, this risk was not significant. Effect size distribution analysis revealed high heterogeneity for age at weaning, number of piglets weaned, piglet weight at weaning, and 21-d adjusted pig wean weight (*I*^2^*P* < 0.043).

### Effect of PG Given to Sows on Progeny Performance After Weaning

The eight comparisons included in this analysis involved 2,208 piglets in total. Inclusion of PG in the sow’s diet varied from 1 to 2 kg/metric ton and the duration of the post weaning phase ranged from 29 to 42 d. Weaning weight was similar between the groups at approximately 6.2 kg/pig (*P* = 0.220). The effects of PG when fed to sows on progeny performance during the postweaning phase are given in Table 4. Forest plots are detailed in Supplementary Appendix 2.

**Table 4.** Effect of *P. guilliermondii* yeast postbiotic fed to sows on piglet performance after weaning^*^

	Control		Effect size calculation				Heterogeneity	
	Mean	SEM	RMD	SE	(95% CI)	*P*-value	*I* ^*2,*^ ^†^	*P*-value
Weaning weight, kg	6.20	0.05	−0.08	0.06	(−0.23; 0.07)	0.220	31.2	0.194
ADG, g/d	344.2	28.6	18.9	8.7	(1.8; 35.9)	0.030	68.97	<0.001
Final weight, kg	18.67	1.30	0.47	0.30	(−0.13; 1.06)	0.124	44.4	0.063
Mortality,^‡^ %	7.65	2.67	0.73	0.16	(0.53; 1.00)	0.055	0	0.412

^*^A total of 8 trials were used in this meta-analysis to determine the effects of feeding sows a product containing *P. guilliermondii* yeast postbiotic (PG; Citristim, ADM Animal Nutrition, Quincy, IL) during gestation and lactation on progeny performance after weaning.

^†^ *I* ^2^ can be interpreted as the proportion of total variation observed between the trials attributable to differences between trials rather than sampling error (chance).

^‡^For the effect size calculation the odd’s ratio is reported.

RMD, raw mean difference; SE, standard error; 95% CI, 95% confidence interval; ADG, average daily gain.

Compared to the CON group, piglets born from sows fed PG had a greater ADG after weaning (+18.9 g/d; *P* = 0.030), a numerically heavier end weight although not statistically different (+470 g; *P* = 0.124), and a tendency for a lower mortality rate postweaning (−27%; *P* = 0.055). High heterogeneity was present for ADG only (*I*^2^*P* < 0.001). The Trim and Fill method did not highlight any missing studies to indicate any risk of publication bias for progeny performance after weaning.

## Discussion

The objective of the present work was to confirm whether PG supplementation to sows could improve their fecundity and offspring performance. Beneficial effects of supplementing sows with yeast-based products during gestation and lactation on farrowing performance has also been described with strains like *S. cerevisiae* (Kim et al., 2008; Hasan et al., 2018). The present meta-analysis confirms a positive impact on litter size at birth when *P. guilliermondii* (PG) yeast is fed to sows. This improvement is highly consistent; however, this has not been described for any other yeast-based products available to the animal nutrition sector. Due to dietary treatments beginning primarily at breeding, it is logical to assume similar ovulation rates between PG and CON groups. During gestation, there are three periods of conceptus loss: implantation before day 30, days 30-40, and days 90-114 (Ford et al., 2002). The last two periods are defined by uterine capacity being limited which induces space as well as nutrient competition for the whole litter. It is unclear how implantation or uterine capacity is influenced by PG.

Kim et al. (2008) fed sows a *S. cerevisiae* yeast culture in gestation and although they did not observe a difference in litter size at birth, they did observe an increased litter wean weight when sows were fed the yeast culture. Lu et al. (2019) fed a live *S. cerevisiae* yeast in gestation and lactation and observed no differences in birth weight, weaning weight, litter size, or preweaning mortality.

Feeding PG to sows during lactation seemed to have only a limited effect until weaning, with no difference in litter weaning weight. However, feeding sows with this product improved growth performance of piglets after weaning. This effect on nursery ADG was in agreement with results from Lu et al. (2019) who fed a live *S. cerevisiae* yeast in gestation and lactation. Other references reported limited or no effect of using *S. cerevisiae* based products with the same procedure on piglets after weaning (Shen et al., 2017; Duan et al., 2019).

Heat killed *P. guilliermondii* was shown to have a potent immune modulating effect, mediated primarily by N-linked mannans (Navarro-Arias et al., 2016). Navarro-Arias et al.(2016) demonstrated that heat inactivated *P. guilliermondii* could activate cytokine production by human peripheral blood mononuclear cells, and more specifically interleukin-6 (IL-6). In another study, the increase of maternal IL-6 production following an infection could induce changes in the intestinal cells of the fetus in mammals, promoting long-term tissue specific fitness of the progeny (Lim et al., 2021). Given the specific structure of mannans in *P. guilliermondii* that constitutes PG, it may be hypothesized that the effect observed on the progeny after weaning when feeding PG to the sows during gestation could be driven by fetal imprinting before birth. More research would be relevant to confirm this hypothesis, and to investigate if a lower inclusion dose would be as efficient. Identifying if the product is efficient only in gestation, or if supplementation during gestation and lactation is needed would be an asset to have more precise information on how to best apply PG in practice.

From birth until weaning, the Trim and Fill method highlighted missing studies for all outcomes except litter birth weight, % light piglets, piglet weight at weaning, wean age, and 21-d adjusted litter weight. Adjustment for risk of publication biases using this method did not affect the overall results, except for number of piglets after cross-foster. In practice, cross-fostering aims to balance the number of piglets per sow across the available cohort sows. This practice is an imposed intervention and is not biologically driven. Adjustment for risk of publication bias would result in similar litter size after cross-foster, in line with common practices. However, in commercial operations, all sows would be fed the same diet, either with or without PG, and any benefit of PG use over nonuse would automatically be transposed into more piglets post-cross-foster and at weaning.

Consequently, in the present case, the difference in litter size before and after cross-foster between PG and CON was reduced due to management practices and did influence the magnitude of the effect of PG on litter size at weaning. In that context, adjusting the number of piglets weaned per sow, considering offspring mortality during the suckling period, would be a more accurate estimation of the potential effect of the PG supplementation.

**Figure 2.**
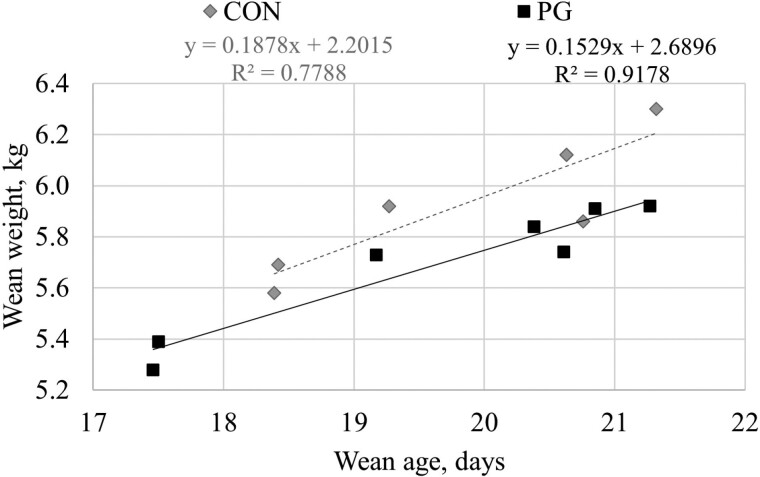
Linear regression of piglet wean weight as function of wean age for control (CON) and *P. guilliermondii* (PG) groups. There was a positive linear correlation between wean weight and wean age with similar (*P* = 0.61) regression equations between the two experimental groups.

Compared to the CON group, PG group exhibited a lighter piglet weaning weight. However, this may be due to a younger age at weaning. This observation is not caused by a direct effect of the product, but by practical management constraints on farms. A regression analysis confirmed the positive linear correlation between wean weight and wean age, and the similarity in the regression equations between the two experimental groups (Figure 2; *P* = 0.61).

The high heterogeneity observed for weaning age and litter size post cross-foster is likely not attributable to PG supplementation. This is likely due to various routine procedures across trial locations. The reason for high heterogeneity of the effect of PG on ADG during the nursery phase is unknown.

In conclusion, the present meta-analysis confirms a consistent beneficial effect of feeding PG to sows during gestation on litter size at birth. Pigs from sows fed PG in gestation and lactation also grew faster post weaning, which could be attributed to fetal imprinting of the immune system before birth.

## Supplementary Material

txae137_suppl_Supplementary_Appendix
