# Supplementary material for: Sow reproductive and progeny growth performance when fed Pichia guilliermondii yeast postbiotic: systematic review and meta-analysis
Source: Transl Anim Sci. 2024 Sep 14;8:txae137. doi: 10.1093/tas/txae137 (PMC11457054; doi:10.1093/tas/txae137)
Supplement: txae137_suppl_Supplementary_Appendix [file txae137_suppl_supplementary_appendix.docx]

**Appendix 1:** Forest plots of meta-analysis – Sow performance until weaning

|  |  |
| --- | --- |
|  |  |
|  |  |
|  |  |
|  |  |
|  |  |
|  |  |

**Appendix 2:** Forest plots of meta-analysis – Nursery performance of offspring from sows included in experimental groups

|  |  |
| --- | --- |
|  |  |
